# MECHANISMS OF BRAIN REJUVENATION

**DOI:** 10.1093/geroni/igz038.2177

**Published:** 2019-11-08

**Authors:** Saul Villeda

**Affiliations:** University of California San Francisco, San Francisco, California, United States

## Abstract

A growing body of work has shown that systemic manipulations, such as heterochronic parabiosis and young blood administration, can partially reverse age-related cellular impairments and loss of cognitive faculties in the aged brain. These studies have revealed an age-dependent bi-directionality in the influence of the systemic environment indicating anti-aging factors in young blood elicit rejuvenation while pro-aging factors in old blood drive aging. It has been proposed that introducing anti-aging factors or mitigating the effect of pro-aging factors may provide effective strategies to rejuvenate aging phenotypes. Despite this potential, much is unknown as to the systemic and molecular mechanisms regulating anti-aging and pro-aging effects of blood-borne factors. I will discuss work from my research group that begins to provide mechanistic insight into the systemic and molecular drivers promoting rejuvenation in the aging brain.

